# PET/CT in Radiotherapy Planning for Head and Neck Cancer

**DOI:** 10.3389/fonc.2012.00189

**Published:** 2012-12-10

**Authors:** Katie Newbold, Ceri Powell

**Affiliations:** ^1^The Royal Marsden National Health Service TrustLondon, UK

**Keywords:** head and neck cancer, PET-CT, biological target volume, radiotherapy, biomarkers

## Abstract

The use of PET/CT as an adjunct in radiotherapy planning is an attractive option in head and neck cancer (HNC) for several reasons. First, with potentially better identification of the disease extent, i.e., staging, the risk of geographical miss of radiation delivery to the gross tumor volume is reduced. Second, in characterizing the biological behavior of the disease for example, areas of hypoxia, rich or poor vascularity, or high cell proliferation, PET/CT can identify biological target volumes either for escalation of radiation dose or to predict the requirement for the addition of a radiosensitizer or alternative treatment strategies. ^18^F-FDG is the most common tracer used in oncology studies, but many other tracers have been investigated with several entering clinical practice, although these remain predominantly in the research domain in HNC.

## Targeting Hypoxia

PET/CT imaging can provide a spatial map of the intra-tumoral distribution of hypoxia before and during radiotherapy using^18^F-fluoromisonidazole (^18^F-FMISO), a nitroimidazole PET tracer that is reduced and bound to cell constituents under hypoxic conditions. ^18^F-FMISO has been shown to be both a predictive and prognostic biomarker in head and neck cancer (HNC). Several studies have shown that the level of hypoxia depicted by ^18^F-FMISO PET before treatment correlates with loco-regional failure (Eschmann et al., 2005; Thorwarth et al., 2005; Rischin et al., 2006). In all tumor types evaluated, poorer outcome (local control and development of metastases) was observed when a significant fraction of the pO_2_ readings were below 7–10 mm Hg (Krohn et al., 2008). Tumor retention of ^18^F-FMISO reflects tissue pO_2_ in the radiobiologically relevant range of 1–10 mm Hg and shows moderate to high correlation with pO_2_ levels measured with Eppendorf electrodes (Gagel et al., 2004). ^18^F-FMISO PET has also been used to define a hypoxic biological target volume (BTV) and theoretical planning studies have demonstrated the potential to dose-escalate to this sub-volume (Rajendran et al., 2006; Thorwarth et al., 2007; Lee et al., 2008). Lee at al. planned a boost of 84 Gy to the hypoxic gross tumor volume (GTV; delineated as areas of ^18^F-FMISO uptake within the ^18^F-FDG-PET/CT GTV) and 70 Gy to the GTV, in 10 patients without exceeding normal tissue tolerance (Lee et al., 2008). An attempt to deliver 105 Gy to the hypoxic GTV was only successful in one of two patients with normal tissue sparing. In a further planning study, Thorwarth et al. (2007) calculated a potential increase in tumor control probability from 55.9 to 70.2% by dose-escalating to an ^18^F-FMISO delineated hypoxic volume in 13 patients using a dose painting by numbers (DPBN) strategy compared with a conventional IMRT plan maintaining iso-toxicity. To date, no clinical studies have been published with outcome of dose escalation to ^18^F-FMISO-defined volumes. One of the limitations of ^18^F-FMISO imaging in this setting may be its temporal variability. Nehmeh et al. (2008) have shown that when patients underwent two baseline ^18^F-FMISO scans 3 days apart, a voxel by voxel analysis of putative hypoxic areas revealed a strong correlation across the two time points in less than half of patients.

An alternative PET tracer for imaging hypoxia is Cu-diacetyl-bis(N4-methylthiosemicarbazone) (Cu-ATSM) which can be labeled with ^60/61/62/64^Cu isotopes with varying physical and production properties. The ^64^Cu isotope presents the best compromise between adapted physical properties and good production yield and is showing promise as a non-invasive marker of tumor hypoxia (Bourgeois et al., 2011). In both normoxic and hypoxic cells the [Cu(II)-ATSM] complex is reduced by intracellular thiols resulting in an unstable [Cu(I)-ATSM] complex. In normoxic cells it is re-oxidized to a stable [Cu(II)-ATSM] complex and diffuses out of cells. Under hypoxic conditions, however, the [Cu(I)-ATSM] slowly dissociates and copper is irreversibly trapped by intracellular copper chaperone proteins. The uptake of ^60^Cu-ATSM has been shown to correlate with tumor pO_2_ in rat models (Lewis et al., 1999). In a feasibility study, Chao et al. (2001) demonstrated a heterogeneous distribution of ^60^Cu-ATSM within the GTV of patients with HNC and planned 80 Gy in 35 fractions to the ATSM-avid tumor sub-volume with 70 Gy in 35 fractions to the GTV without compromising normal tissue constraints.

## Imaging Proliferation

Accelerated repopulation during radiotherapy for HNC is another mechanism of radioresistance which adversely affects outcome. PET tracers which seek to image DNA synthesis have been developed and may offer an advantage in specificity over ^18^F-FDG-PET which is also taken up by peri-tumoral inflammatory cells. 3′-Deoxy-3′-^18^F-fluorothymidine (^18^F-FLT) is a tracer that reflects the activity of thymidine kinase 1, a key enzyme in DNA synthesis (Shields et al., 1998) and is taken up by dividing tumor cells but not by terminally differentiated immune response cells. Troost et al. have evaluated the role of ^18^F-FLT in early response assessment to radiotherapy in 10 patients with HNC (two received concomitant chemotherapy). Patients underwent ^18^F-FLT PET/CT scan before and during the second and fourth weeks of radiotherapy. The GTV delineated on CT decreased significantly in the fourth week but not in the initial phase of treatment whereas significant changes in SUV_max_ and SUV_mean_ were observed on ^18^F-FLT images as early as 1 week into radiotherapy and decreased even further before the fourth week of treatment. An arbitrary, fixed threshold (80%) of the SUV_max_ was defined such that a tumor sub-volume could be delineated in at least the first and second ^18^F-FLT PET scans. Using this 80% isocontour, dose escalation was demonstrated to be technically feasible, although with only a modest dose increase (68–74 Gy) in a small sub-volume and this was only attempted in one patient. Accelerated repopulation is thought to occur after 4 weeks of radiotherapy but this is not reflected in ^18^F-FLT PET uptake during radiation in studies in HNC. Studies in radiation for squamous cell carcinoma (SCC) of the esophagus also showed reducing ^18^F-FLT PET uptake as the duration of radiation increased. However, interestingly in patients who experienced a treatment break, increased uptake of ^18^F-FLT PET was observed after treatment interruptions which may reflect accelerated repopulation (Yue et al., 2010).

As hypoxic and proliferative tumor sub-volumes diminish during treatment, it is likely that radiation boosts targeted to this area would need to be delivered early in the treatment course in order to maintain the temporal and spatial accuracy of such a boost. This may be best delivered by an up-front boost rather than a simultaneous integrated boost for the duration of treatment though further studies are required to address this question.

## Incorporating PET/CT into Radiation Planning and Delivery

Although planning radiation delivery to a PET-defined BTV is an attractive strategy, there are uncertainties relating to the accuracy of PET/CT in this setting. The potential advantages and disadvantages are summarized in Table 1. One of the main areas of uncertainty relates to segmentation of the PET target volume, i.e., definition of the ‘edge’ of the target. Five main methods have been developed. (1) Visual interpretation, which is highly operator-dependent and susceptible to window-level settings and interpretation differences (Nishioka et al., 2002; Heron et al., 2004; Riegel et al., 2006). (2) Isocontouring based on a fixed standardized uptake value (SUV). (3) Fixed threshold of maximum tumor signal intensity (40 or 50%) (Ciernik et al., 2003; Nestle et al., 2005; Paulino et al., 2005). A major disadvantage of using the maximum tumor SUV is that it is highly dependent on contrast recovery and noise properties that vary by scanners and reconstructive protocols, therefore, superior techniques have been sought. (4) Variable threshold based on adaptive signal-to-background ratio (SBR) (Daisne et al., 2004). (5) Iterative background-subtracted relative-threshold using watershed transformation and hierarchical cluster analysis in which the optimal relative-threshold depends on the lesion size not the SBR (Geets et al., 2007a,b; van Dalen et al., 2007).

**Table 1:** **Summary of potential advantages and disadvantages to the use of ^18^FDG-PET in radiotherapy planning for head and neck cancer**.

Advantages	Disadvantages
May reduce inter-observer variation in GTV delineation (Ciernik et al., 2003; Riegel et al., 2006)	Limited spatial resolution
Reduces size of GTV (Daisne et al., 2004)	Lack of standardized method for signal segmentation
Identify tumor or LN missed by CT/MRI	False positive PET readings due to inflammation
Identify parts of GTV potentially requiring additional radiation dose	

The first four of these methods have been compared in a planning study of 78 patients which found that both the volume and shape of GTV was influenced by the segmentation method (Schinagl et al., 2007). The visual method volumes were close to CT-defined GTV and all automated volumes were smaller. A fixed SUV of 2.5 failed to identify the GTV in 45% of cases and in 29–64% of patients more than 20% of PET-based GTV was outside the clinical/CT-based GTV although it is unknown if this represents a false positive due to peri-tumoral inflammation. A threshold of 50% maximum SUV (SUV_max_) produced a GTV volume most similar to the SBR method which has been shown to be more similar to pathological volumes than CT or MRI. All three modalities, however, failed to identify superficial tumor extension due to a lack of spatial resolution (Daisne et al., 2004). Although the gradient-based segmentation method has been shown to be more accurate than the SBR method when compared with macroscopic extent of resected laryngeal tumors, neither of these techniques are widely available outside the center where they were developed (Geets et al., 2007a,b).

Moule et al. (2010) compared functional volumes delineated by SUV cut off (2.5, 3.0, 3.5, and 4.0 bodyweight g/ml) and percentage of the SUV_max_ (30, 35, 40, 45, and 50%) thresholds on ^18^F-FDG-PET scans acquired at 0, 10, 44, and 66 Gy of radiation. Using the SUV_max_ method, the software was unable to differentiate effectively between tumor and background uptake after 36 Gy and the delineated volumes increased as the delivered radiation dose increased. In a planning study based on the same dataset using an adaptive iterative algorithm weighted according to the mean SUV within the ROI to delineate target volumes, no significant reduction in the primary target volumes was observed during radiotherapy (Moule et al., 2011).

Due to the sub-optimal outcome for a significant proportion of patients with HNC, PET-defined BTVs are an attractive target for dose escalation. The dose required to overcome radioresistance within the target volume is unknown. Radiobiological modeling may predict such doses, however, current treatment algorithms in the head and neck region are already close to patient tolerance. Two clinical studies have been published evaluating the outcome of ^18^F-FDG-PET-based dose escalation. In 2007, Madani et al. (2007) reported a phase 1 trial boosting to two planned dose levels of 25 and 30 Gy delivered in 10 fractions followed by 22 fractions of 2.16 Gy using IMRT (total dose 72.5 or 77.5 Gy in 32 fractions). Twenty-three patients were enrolled at dose level 1 and 18 at level 2. Two cases of dose-limiting toxicity occurred at dose level 1 (Grade 4 dermatitis and Grade 4 dysphagia) and a treatment-related death at dose level 2. Despite the doses achieved and toxicity experienced, in four of nine patients, the site of relapse was within the boosted ^18^F-FDG-PET-delineated region. More recently, the same group has reported results of a phase 1 trial of DPBN delivering a mean dose of 80.9 Gy to the high dose CTV (seven patients) or 85.9 Gy to the GTV (14 patients) in 32 fractions (Duprez et al., 2011). For each patient the first 10 fractions of treatment comprised a voxel intensity-based (DPBN) IMRT plan using a baseline ^18^F-FDG-PET scan, fractions 11–20 used the same technique based on an ^18^F-FDG-PET scan acquired after the eighth fraction and the remaining fractions were delivered using a uniform dose IMRT plan. The adaptive planning reduced the irradiated volume of both target and normal tissue, particularly in dose level 2 where dose was escalated to GTV. There was no grade 4 acute toxicity, however, only 9 out of 21 patients received concomitant chemotherapy and further follow-up has identified mucosal ulcers as the dose-limiting toxicity (Madani et al., 2011). Six cases of mucosal ulcers were observed at a latency of 4–10 months following treatment, of which five were observed at dose level II (median total dose of 85.9 Gy to the GTV). This led to the establishment of dose level I (median total dose of 80.9 Gy to the high dose CTV) as the maximum tolerated dose in that trial and highlights the importance of longer-term follow-up in these studies.

## ^18^F-FDG-PET as a Biomarker in HNC

In addition to a role in target definition, ^18^F-FDG-PET imaging can identify patients who are less likely to respond to current treatment algorithms and may benefit from alternative treatments, dose escalation, or early salvage options. Several published studies have demonstrated this role using baseline ^18^F-FDG-PET parameters (Inokuchi et al., 2011; Higgins et al., 2012; Liu et al., 2012) or ^18^F-FDG-PET imaging acquired 2–4 months following completion of treatment (Yao et al., 2005; Krabbe et al., 2009; Gupta et al., 2010; Moeller et al., 2010; Ceulemans et al., 2011), Table 2. In a study by Higgins et al. a higher baseline SUV_mean_ in a cohort of 88 patients was predictive of DFS (*P* = 0.01), Table 2 (Higgins et al., 2012). Patients with a SUV_mean_ below 7 (median value for the cohort) had a prolonged 2 years DFS (82 vs. 58%, *P* = 0.03) compared with those with a baseline SUV_mean_ above 7. Neither SUV_max_ in the primary tumor or LN or total lesion glycolysis (SUV_mean_ × tumor volume) was prognostic for any of the clinical endpoints evaluated. In contrast, in a larger study, Inokuchi et al. (2011) found baseline SUV_max_ to be predictive of outcome with a nodal SUV_max_ ≥ 6 predicting for a poorer 3 years DFS (44 vs. 69% *P* = 0.004). Additionally, in the group with a nodal SUV_max_ ≥ 6, those undergoing a planned neck dissection following CRT had a more favorable outcome than those who were observed (*P* = 0.04). Both these studies included patients with all sub-types of HNC.

**Table 2:** **Summary of studies evaluating ^18^F-FDG-PET as a predictor of response to CRT in patients with HNC**.

Author	*n*	^18^F-FDG-PET	Diagnostic accuracy for residual disease (%)					Survival prediction
			Sensitivity	Specificity	PPV	NPV	Accuracy	
Gupta et al. (2010)	57	9 weeks PT	Primary site					LRC and OS
			50	91.8	50	91.8	86	
			Neck				
			62.5	98	83.3	94.1	93	
Ceulemans et al. (2011)	40	47 Gy	28.6	81.8	80	31	42.5	None
		4 months PT	78.6	75	88	60	77.5	OS
Krabbe et al. (2009)	48	3, 6, 9, 12 month PT	100	43	51	100		
McCollum et al. (2004)	40	After ICT	100	65	27	100	69	
		After CRT	67	53	46	73	58	
Moeller et al. (2010)	98	8 weeks PT	Primary site					SUV_max_ and OS SUV_max_ ≥ 6
			50	85	50	92		
			Lymph nodes					
			25	50	6	85		
Yao et al. (2005)	53	15 weeks PT	100	94	43	100		
Hentschel et al. (2011)	37	10–20 Gy						Δ_SUVmax10/20_ ≥ 50% predicts 2 years OS
Higgins et al. (2012)	88	Baseline						SUV_mean_ and DFS
Inokuchi et al. (2011)	178	Baseline						SUV_max_ and DFS, NPFS, DMFS
Liu et al. (2012)	75	Baseline						SUV_max_ and 5-years DFS, LFFS
Yoon et al. (2011)	21	After ICT						SUV_max_ < 4.8, or ↓65% and CR

*PPV, positive predictive value; NPV, negative predictive value; *n*, number of patients in study; LRC, loco-regional control; OS, overall survival; PT, post-treatment; ICT, induction chemotherapy; SUV_mean_, mean standardized uptake value; SUV_max_, maximum SUV; DFS, disease-free survival; NPFS, nodal progression-free survival; DMFS, distant metastasis-free survival; LFFS, local failure-free survival*.

Monitoring of early response during treatment could allow treatment modification or adaptation and this has been shown in a proof-of-principle study by Geets et al. (2007a). Ten patients with pharyngo-laryngeal SCC treated with CRT were subjected to CT, MRI, and ^18^F-FDG-PET during treatment (at baseline and after mean prescribed doses of 14, 25, 35, and 45 Gy). Throughout the course of radiotherapy, GTVs. delineated (using a gradient-based method) on ^18^F-FDG-PET substantially decreased and were always smaller than those defined with CT and MRI (*P* < 0.001). During treatment this resulted in a progressive reduction of irradiated volumes by 15–40% compared with standard CT-based volumes delineated pre-treatment. This adaptive approach only impacted the high dose volumes (≥V_90_) with little additional sparing of organs at risk (OAR).

The optimum timing of ^18^F-FDG-PET imaging following treatment is uncertain due to ^18^F-FDG uptake in non-malignant inflammatory tissue which complicates interpretation. In a study by Greven et al., 45 patients with HNC underwent ^18^F-FDG-PET scans at 1, 4, 12, and 24 months following radiotherapy for HNC. Specificity for detection of residual or recurrent tumor was high at both 1 and 4 months (95 and 90%, respectively), however, sensitivity increased from 59% at 1 month to 100% at 4 months suggesting the optimum time for post-treatment assessment is between 1 and 4 months (Greven et al., 2001; Bussink et al., 2010). Encouraging results have recently been published for ^18^FDG-PET in response assessment following definitive chemoradiotherapy (CRT) in predicting loco-regional control and improved survival in those with a negative scan (at a median of 9 weeks post-CRT) (Gupta et al., 2010), and in several other studies, Table 2.

Ceulemans et al. (2011) have evaluated whether an ^18^F-FDG-PET scan during treatment (after 47 Gy) could replace one at 4 months post-treatment as a predictive and prognostic biomarker. Both PET scans had a high specificity and PPV, however, the sensitivity of the post-treatment scan was much higher than during treatment (79 vs. 29%). Additionally there was a significant difference in overall survival between patients with a complete response vs. a non-complete response on PET scanning at 4 months (92 vs. 50%) but not after 47 Gy. In contrast, an early reduction in SUV_max_ during treatment may be predictive of outcome. Hentschel et al. (2011) evaluated serial ^18^F-FDG-PET scans at three time points during CRT comparing changes in SUV with baseline. In their series of 37 patients a reduction of SUV_max_ of ≥50% after 10–20 Gy of radiation (Δ_SUVmax10/20_ ≥ 50%) was predictive of 2 years OS (88 vs. 38%, *P* = 0.02), and a below median volume of disease defined on baseline PET was also predictive of 2 year OS (83 vs. 34%, *P* = 0.02).

## PET/CT in the Context of Induction Chemotherapy

The majority of published studies have been undertaken in patients receiving definitive CRT alone. As induction chemotherapy (IC) is increasingly used, it is unclear how applicable these PET-based response prediction data are for this population. Only two studies have evaluated the role of ^18^F-FDG-PET imaging in HNC patients undergoing IC in addition to CRT. In the first, McCollum et al. (2004) evaluated 33 patients undergoing ^18^F-FDG-PET imaging following IC and 37 with imaging 4–12 weeks after completion of CRT. Twenty-six patients had a repeat biopsy after IC against which the^18^F-FDG-PET findings were compared. ^18^F-FDG-PET was found to have a high sensitivity and moderate specificity in the setting but these findings were not related to outcome. The overall accuracy of PET for predicting persistent tumor at the primary site after IC was 69% (95% CI, 51–87%) and NPV 100% (95% CI, 78–100%), Table 2. In the second study, Yoon et al. showed that a SUV_max_ of 4.8 on interim ^18^F-FDG-PET could predict clinical complete response after CRT (100 vs. 20%, *p* = 0.001), PFS (median, not reached vs. 8.5 months, *p* < 0.001), and OS (median, not reached vs. 12.0 months, *p* = 0.001). A 65% decrease in SUV_max_ after IC from baseline in the same study could also predict complete clinical response after CRT (100 vs. 33.3%, *p* = 0.003), PFS (median, not reached vs. 8.9 months, *p* < 0.001), and OS (median, not reached vs. 24.4 months, *p* = 0.001). Of note, only patients with a partial response to IC as determined by Response Evaluation Criteria In Solid Tumors (RECIST) were included in this study.

**Figure 1:**
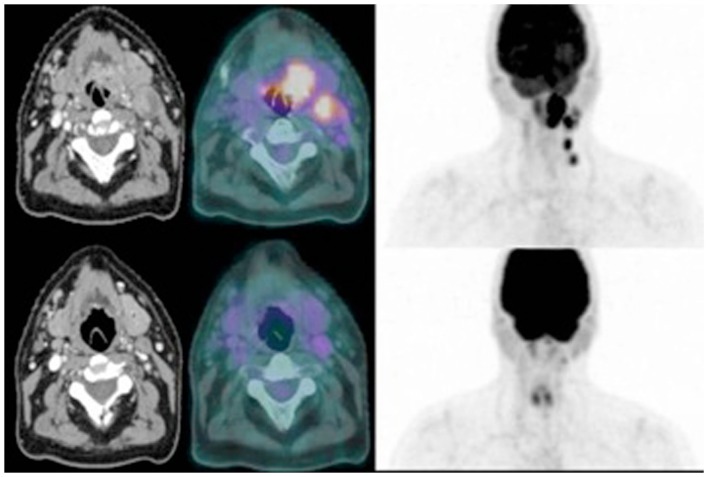
**Comparative axial images from contrast-enhanced CT (left), and ^18^F-FDG-PET/CT (right) for a patient with T2N2b SCC tonsil before (top) and after (bottom) induction chemotherapy**.

Our own experience has shown that ^18^F-FDG-PET can alter the conventionally defined GTV and clinical target volume (CTV) (Newbold et al., 2008). A subsequent feasibility study is in progress investigating how tumor metabolism changes during treatment with IC followed by CRT and whether ^18^F-FDG-PET imaging is able to identify a relevant BTV which may benefit from treatment intensification strategies. Patients undergo standard anatomical CT and MRI and functional imaging with ^18^F-FDG-PET/CT, pre-IC, post IC, and at 3 and 6 months post completion of treatment. It was initially anticipated that it would be possible to delineate a BTV on both functional and anatomical imaging at the first two time points as a minimum. A significantly larger reduction in metabolic signal than in the volume defined on CT or anatomical MRI sequences has been observed which may imply that ^18^F-FDG-PET is a more sensitive method for assessing early response to treatment than conventional anatomical imaging. However, initial analysis shows that the biological response to IC (Figure 1) is so marked that it will be difficult, or even impossible, to delineate small volume residual disease during radiotherapy planning. In the majority of patients a metabolic signal is no longer detected on ^18^F-FDG-PET/CT images following IC. This may represent stunning and a false negative due to metabolic switch off, but this is unlikely as all the ^18^F-FDG-PET images were acquired more than 10 days after administration of chemotherapy. It does though, have significant implications for image-guided radiotherapy strategies that aim to deliver radiation boosts to BTV defined on functional imaging. Based on these early observations, such a boost volume would have to be defined on the original baseline images acquired before the initiation of IC. The physical and biological relevance of this BTV at the start of CRT (following 6 weeks of IC) is far from clear.

## Limitations of PET/CT Imaging in Radiation Planning

Functional imaging and its application to radiotherapy is a rapidly expanding field with new modalities and techniques constantly developing and evolving. As technologies improve, so it will be important to pay careful attention to their implementation. It is crucial, therefore, that good collaborative links are maintained with diagnostic physicians in order to assure correct interpretation of the functional imaging acquired. This will be particularly relevant as scanners are incorporated into RT departments and images acquired solely for RT planning purposes. It may also become necessary to develop improved quantitative, rather than qualitative, assessment of the response to treatment. For example, the concept of a ‘metabolic response to treatment’ where the criteria of a change >1 SUV and a 20–30% relative change in SUV are both met is less likely to be a chance finding than either criterion alone (de Langen et al., 2012).

Another important limitation is the finite resolution of PET. Biological image-guided radiotherapy aims at specifically irradiating biologically relevant sub-volumes within the tumor and requires functional imaging to be sensitive and specific enough to image the biological pathway of interest such as tumor metabolism. Preclinical studies have shown discrepancies between imaging with a small-animal PET scanner with a spatial resolution of 2.7 mm and the underlying microscopic reality represented by autoradiography (Christian et al., 2009). Such a discrepancy means the macroscopic assessment of tumors with molecular imaging might not necessarily reflect their microregional distribution (Bussink et al., 2010). Additionally, many microregional tumor areas are likely to co-exist within one PET voxel, and have varied exposure to acute and chronic hypoxia. Negative scan findings cannot, therefore, exclude the presence of microscopic tissue involvement, and precise anatomic localization of the signal can be difficult in certain anatomic regions (Fletcher et al., 2008). As the set-up tolerance is up to 3 mm for patients with HNC immobilized in TP masks, caution should also be given to dose prescriptions to a voxel of approximately 5 mm size as its precise location intra- or inter-fraction cannot be assured. Heterogeneous dose prescriptions varied by voxel may not be possible with current delivery systems. Furthermore, the microenvironment of treated and untreated tumors changes with time. This has implications for signal validation with repeat imaging and more work is required in this area to quantify this uncertainty particularly in the use of functional imaging for DPBN.

**Figure 2:**
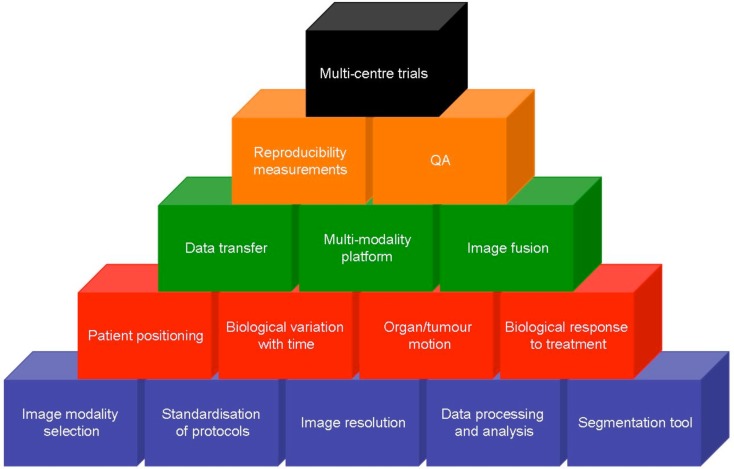
**Complexity of implementing functional imaging into the management of head and neck cancer**. Each building block represents a challenge to be overcome in order to validate promising data and perform successful multi-center trials. Data acquisition (blue) needs to be standardized before the influence of biological factors (red) can be interpreted. Data transfer and widely available multi-modality viewing platforms (green) need to be developed with rigors QA and robust data (orange) before ultimately, multi-center trials can be undertaken.

Despite a decade of activity in this field and thousands of peer-reviewed publications demonstrating the utility of molecular and functional imaging, this is yet to be implemented into routine clinical practice. The reason for this is multi-factorial and relates to much of the data being generated from single institutions series with variations in imaging modality, sequence acquisition, data processing, and analysis tools. The next challenge is implementing PET imaging and tackling the associated uncertainties, Figure 2. As preliminary findings are validated in larger studies, so attention to standardization of protocols and image processing and data analysis must occur. This is necessary not only for implementing findings from studies performed at other institutions but, most importantly, in the design of multi-center trials which must include rigorous QA (quality assurance). For example, metabolic treatment volumes are often defined in relation to SUV_max_, which is highly dependent on contrast recovery and noise properties that vary across scanners and reconstructive protocols. The European association of nuclear medicine (EANM) have published guidelines in an attempt to standardize PET image acquisition, processing and reporting, however, these are yet to be fully adopted (Boellaard et al., 2010). Thresholds for metabolic change using ^18^F-FDG-PET are approximately −34 to +52% for individual centers and −26 to +39% after centralized QA (Velasquez et al., 2009). Hence there is a need for standardization in relation to the use of SUVs and SUV changes in studies of treatment response assessments (Boellaard, 2011).

## Conclusion

It is likely that PET/CT as a modality with multiple tracers will continue to have a role in determining treatment strategies for HNC. Defining BTVs to target for radiation dose escalation will only be one aspect with tumor phenotyping using PET/CT parameters as biomarkers another, providing methods of stratification of patients based on likelihood of response at baseline assessment.

## Conflict of Interest Statement

The authors declare that the research was conducted in the absence of any commercial or financial relationships that could be construed as a potential conflict of interest.
